# Chronic Appendicitis: Possible Differential Diagnosis in Patients with Chronic Abdominal Pain

**DOI:** 10.1155/2024/6032042

**Published:** 2024-01-09

**Authors:** Iva Ljubas, Ivana Jurca, Dora Grgić

**Affiliations:** ^1^Department of Emergency Medicine, University Hospital Centre Zagreb, Croatia; ^2^Department of Interventional Radiology, University Hospital Centre Zagreb, Croatia; ^3^Department of Gastroenterology and Hepatology, University Hospital Centre Zagreb, Croatia

## Abstract

In the emergency department, patients frequently present with abdominal pain, with a variety of different causes ranging from intra-abdominal to extra-abdominal and retroperitoneal pathologies which can affect all age groups. Chronic appendicitis is a rare medical condition characterized by less severe and continuous abdominal pain and a clinical picture lasting longer than 1-2 days and extending over months, even years, and it is not always possible to consider it as a preliminary diagnosis. We represent a case report of chronic appendicitis where the patient's clinical picture led the diagnostics and treatment in different directions and delayed the diagnosis. Namely, our patient was radiologically diagnosed with a collection of denser content retroperitoneally in the right lower quadrant of the abdomen, which in the first place was not related to possible appendicitis, regarding complaints. The existence of chronic appendicitis is a diagnosis unfamiliar to many clinicians and has no official diagnostic criteria. After diagnosis, treatment usually begins with antibiotics, and the next most common step is surgery. The optimal treatment for this condition is unknown. With this case report, we wish to draw attention to chronic appendicitis as a possible differential diagnosis in patients with chronic abdominal pain.

## 1. Introduction

In the emergency department or gastroenterology clinic, patients frequently present with abdominal pain, with a variety of different causes ranging from intra-abdominal to extra-abdominal and retroperitoneal pathologies which can affect all age groups [1, 2]. Acute appendicitis is one of the most common surgical emergencies with the lifetime risk of developing around 7% and usually requires surgical treatment [3]. On the other hand, chronic appendicitis (CA) is a rare medical condition characterized by less severe and continuous abdominal pain and a clinical picture lasting longer than 1-2 days and extending over months, even years. and it is not always possible to consider it as a preliminary diagnosis. Also, the exact etiology of CA is unclear [4].

We present a case report of a patient with chronic appendicitis where the patient's clinical picture led the diagnostics and treatment in different directions and delayed the diagnosis.

There are not many cases described in the literature about chronic hip pain, weight loss, and chronic appendicitis, which is why we consider our case report to be important. Especially in the emergency department, one should be careful and keep in mind that even though such diagnoses are rare, they are possible.

## 2. Patient Information

A 63-year-old Croatian male, previously healthy with no family history of abdominal disease, was administered to the gastroenterology department to rule out possible malignancy due to unintended weight loss of 10 kg in the last month, inappetence, and weakness. The patient was initially examined in the emergency department 4 days earlier with a variety of symptoms including shortness of breath and difficulty with breathing for the last 12 months, weight loss, inappetence, general weakness, and dull pain in his right thigh, especially in the hip area for the last two months, not associated with defecation or urination. The patient was previously healthy and was not taking any chronical therapy, had no history of allergies, and admitted consuming alcohol and cigarettes on a daily basis. Also, he had negative family history of malignancies.

## 3. Clinical Findings

On initial examination in the emergency department, there were no signs of abdominal tenderness or pain in the abdomen and hips and no palpable tumours. He had a leukocyte count of 8.3 × 10^∗^9/*L* (normal range: 3.9–10 × 109/L), C-reactive protein (CRP) (98.3 mg/L) (normal range: <5 mg/L), normocytic anaemia (haemoglobin 91 g/L, MCV 91.9), normal liver-parameters, and slightly elevated D-dimer values (1.26 mg/L). An US of the abdomen and X-ray of the chest and of the right hip and pelvis showed no signs of acute pathology. Additionally, CT pulmonary angiogram was performed but showed no signs of pulmonary embolism. The patient was discharged from the emergency department and was admitted to the gastroenterology department 4 days later for further examinations.

## 4. Diagnostic Assessment

**Figure 1:**
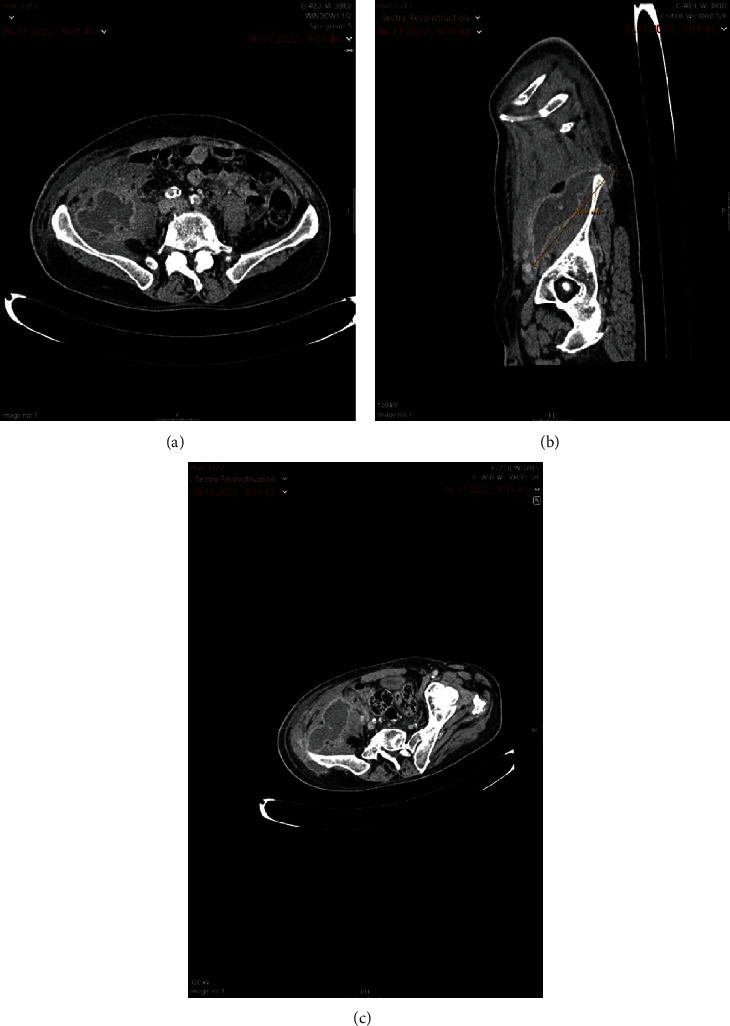
(a–c) Contrast-enhanced computed tomography (CECT) of abdomen and pelvis showing multiobulated, well-marked, and encapsulated dense fluid collection, spreading from pelvic retroperitoneal space to posterior pararenal space and subcutaneous soft tissue above right iliac crest. The collection also spreads into the right iliopsoas and oblique abdominal muscle.

Esophagogastroduodenoscopy was performed on the day of admission, and type I hiatal hernia was found with mild disfunction of the cardiac sphincter. Colonoscopy was not performed due to patients' condition and worsening of symptoms. Additional computed tomography (CT) scan of the abdomen and pelvis was performed on the second day of admission and demonstrated a collection of denser content retroperitoneally in the right lower quadrant of the abdomen, with posterior extension into the subcutaneous fatty tissue and anterior extension to the right iliopsoas muscle to the level of the right femoroacetabular joint. Figures 1(a)–1(c).

## 5. Therapeutic Intervention

The patient was examined by the consultant surgeon and then presented to the abdominal surgery team, and it was agreed that the first therapeutic procedure should be percutaneous abscess drainage, which was done on the second day of admission under CT control. After the procedure, the patient's condition worsened as he developed sepsis, and he was transferred to the department of ICU. When administered to the ICU, the patient was hypotensive, hypoxic, somnolent, and presented with new-onset atrial fibrillation. The patient was treated with symptomatic therapy, amiodaron to perfuse, pantoprazole, and meropenem. After stabilizing the patient's vital signs and arrhythmia, the surgery team was consulted, and acute laparotomic surgery was indicated and done on the third day of admission. Interoperate, freely denser fluid was found and evacuated, and leftovers from the perforated appendix were also removed.

## 6. Follow-Up and Outcomes

11 days postoperative, the patient was discharged home with the recommendation of symptomatic therapy and a follow-up check-up in two weeks at the hospital. Three months after the appendectomy, the patient was symptom-free. All of his laboratory findings were normal, he gained weight, and he no longer suffered from hip pain.

## 7. Discussion

While the exact pathophysiology of acute appendicitis is well known, the existence of chronic appendicitis is a diagnosis unfamiliar to many clinicians and has no official diagnostic criteria. CA mainly presents as a less severe, nearly continuous sharp to dull abdominal pain lasting longer than 2 days, often extending to weeks, months, or even years [5]. Other symptoms of CA include fever, abdominal swelling and tenderness, fatigue or lethargy, malaise, or nausea and diarrhoea. These symptoms most commonly come and go, which can make the disease more difficult to diagnose [6]. The precise ethology of CA is unknown but is likewise believed to be a result of partial or transient obstruction of the appendiceal lumen. It is unknown if CA is an independent disease entity or if is it preceded by an untreated or insufficiently treated acute appendicitis. To conclude, it is hard to differentiate between acute and chronic appendicitis, since the latter is very rare. It is believed that the only, or better to say, main difference between acute and chronic appendicitis is the duration of pain and the clinical picture—the symptoms are often milder than in acute appendicitis, which can lead to misdiagnosis and diagnostic delay [7].

The radiological findings have been estimated to be identical in chronic and in acute appendicitis; by CT, they include pericecal stranding, dilatation appendix, apical thickening, and adenopathy. In some cases, pathological findings have led to the final diagnosis; they include infiltration by lymphocytes, histiocytes and plasma cells in the lamina propria, hyperplasia of lymphoid nodes, and fibrosis [6]. After diagnosis, treatment usually begins with antibiotics and intravenous fluid, and some mild cases can be treated completely like that. The next most common step is surgery appendectomy, which can be laparoscopic or laparotomy. The optimal treatment for this condition is unknown [7].

## 8. Conclusion

With this case report, we wish to draw attention to chronic appendicitis as a possible differential diagnosis in patients with chronic abdominal pain, particularly if it is located in the lower right abdomen and is accompanied with inappetence and weight loss. Radiological imaging with US and/or CT scan can be useful, and further examination must be made in patients who present with similar clinical conditions.

## Data Availability

The data used to support the findings of this study are included within the article.
